# Phytochemistry and Biological Activities of *Murraya* Species

**DOI:** 10.3390/molecules28155901

**Published:** 2023-08-05

**Authors:** Ricky Yohanes, Desi Harneti, Unang Supratman, Sofa Fajriah, Tarso Rudiana

**Affiliations:** 1 Department of Chemistry, Faculty of Mathematics and Natural Sciences, Universitas Padjadjaran, Jatinangor, Sumedang 45363, Indonesia; 2 Central Laboratory, Universitas Padjadjaran, Jatinangor, Sumedang 45363, Indonesia; 3 Research Center for Pharmaceutical Ingredients and Traditional Medicine, National Research and Innovation Agency (BRIN), Complex Cibinong Science—BRIN, Cibinong 16911, Indonesia; 4 Department of Chemistry, Faculty of Sciences Pharmacy and Health, Universitas Mathlaul Anwar, Pandeglang 42273, Indonesia

**Keywords:** *Murraya*, phytochemistry, secondary metabolites, Rutaceae

## Abstract

*Murraya* is a plant genus within the Rutaceae family comprising over 17 species, which are widely distributed in Asia, Australia, and the Pacific Islands. Furthermore, these species have been used in traditional medicine to treat fever, pain, and dysentery. Several reports have also extensively studied the leaves, seeds, stembark, and bark of *Murraya* from 1965 to 2023 to explore their natural product composition. Various phytochemical studies have revealed the isolation of 413 compounds recorded, comprising coumarins, terpenoids, flavonoids, and aromatics, as well as alkaloids, which constitute the largest proportion (46.9%). These isolated compounds have long been known to exhibit different bioactivities, such as cytotoxic and anti-inflammatory properties. Cytotoxic activity has been observed against HCT 116, HeLa, HepG2, and other cell lines. Previous studies have also reported the presence of antifungal, hepatoprotective, antihyperlipidemic, antidiarrheal, and antioxidant effects. Therefore, this review provides a comprehensive overview of *Murraya* species, highlighting their phytochemistry, biological activities, and potential as a source of active natural compounds.

## 1. Introduction

The Rutaceae family comprises over 150 genera that are distributed across the globe. Furthermore, one of these genera is *Murraya*, consisting of 17 species, which are spread across Asia, Australia, and the Pacific region. The ethnobotanical applications of the genus encompass a diverse range of uses, such as the landscaping of construction buildings, and some species can be grafted onto citrus rootstocks. Various plant parts of its members have also been used in traditional medicine to treat fever, pain, and dysentery [1]. 

Phytochemical studies on *Marraya* have been carried out since 1965, with a focus on identifying its potential biological activity. This genus has been investigated for various bioactivities, including cytotoxic [2], anti-inflammatory [3], antihyperlipidemic [4], antidiarrheal [5], and antioxidant [6] activities. In an initial study conducted by Chakraborty et al. [7] in 1965, a carbazole alkaloid-type compound named murrayanine (**98**) was isolated, showing significant anti-inflammatory potential [3]. Furthermore, other isolated alkaloid-type compounds included yuehchukene (**186**) and mahanine (**26**), which exhibited strong anti-implantation [8] and cytotoxic [2] activities, respectively. 

The rapid growth of health problems has necessitated the need for urgent solutions, thereby making bioactive compounds from *Murraya* a starting point for drug development.

Over the course of 56 years, extensive studies have been conducted on the *Marraya* genus, leading to the identification of five classes of metabolites, with alkaloids being the main component. Based on the findings, there are no extensive reports on the phytochemistry and biological activities of the entire genus. Therefore, this review provides a comprehensive overview of *Murraya* species, highlighting their phytochemistry, biological activities, and potential as a source of active natural compounds. The results of this study are expected to serve as a foundation for future studies, which aim to identify chemical content from natural resources and discover new drugs.

## 2. Methodology 

**Figure 1:**
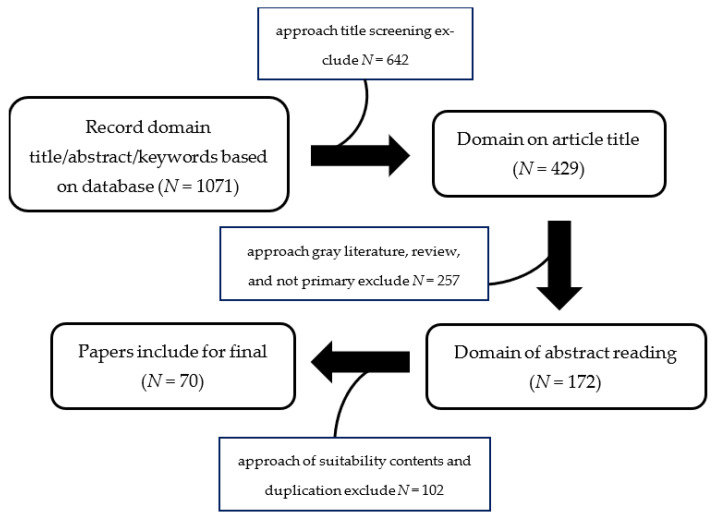
The flow diagram for the systematic review.

This study started with a literature search on *Murraya* species and all the synonyms were confirmed from the plant list (theplantlist.org, [accessed on 20 February 2023]), International Plant Names Index (ipni.org, [accessed on 20 February 2023]), Royal Botanical Gardens (kew.org), [accessed on 20 February 2023]), and tropicos (tropicos.org, [accessed on 20 February 2023]) databases. Furthermore, literature articles were collected from databases such as SciFinder, PubMed, Google Scholar, and Scopus. These articles were filtered based on their abstracts or keywords. A collection of relevant papers published between 1963 and 2023 was then obtained, focusing on the biological and phytochemical properties of *Marraya*. A systematic review was carried out using a flow diagram and meta-analysis studies were gathered from the database search. The identification of relevant papers was carried out with an approach involving title screening, gray literature exploration, review, excluding primary sources, and the removal of duplicate entries. The selected papers were then collected and subjected to further analysis, as shown in Figure 1 [9].

## 3. Botany

**Figure 2:**
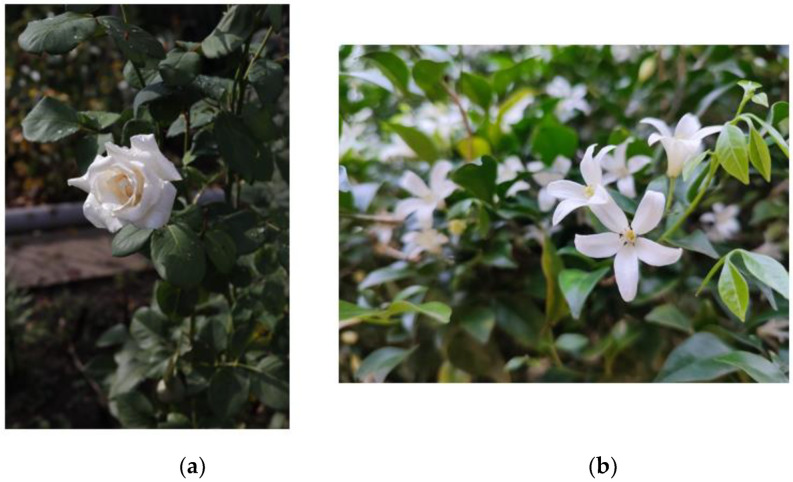
Murraya genus. (**a**) The flower and leaves of *M. paniculata* [15]. (**b**) The flowers and leaves of *M. koenigii* [16].

The plants in the genus *Murraya* were often angiosperms widely distributed in tropical and subtropical regions, including East, Southern, and Southeast Asia, northern Australia, and several areas in South America. The plants were characterized by an average maximum height of 3.5 m, with alternate and odd-pinnate leaves, as well as terminal and/or axillary inflorescence. The seeds typically had a seed coat that could be membranous or fleshy, with straight embryos and elliptic cotyledons. Furthermore, the hypocotyl was partially enclosed between the cotyledons, with four or five petals [1]. One of the *Murraya* species, *Murraya paniculata*, is known as orange jasmine or kemuning in Indonesia and some other countries [10]. Additionally, the species *Murraya koenigii* is widely known as curry tree and the leaves are commonly used as a part of local cuisine in India [11,12,13] (Figure 2). The edibility of this variety of *Murraya* plants has been studied. Liaqat et al. [14], in the research on the toxicology of the Rutaceae family, including *Murraya*, stated that the oil content from *Murraya* is considered safe for internal use with caution. 

## 4. Phytochemistry

### 4.1. Overview of Isolated Compounds from Murraya Species

**Figure 3:**
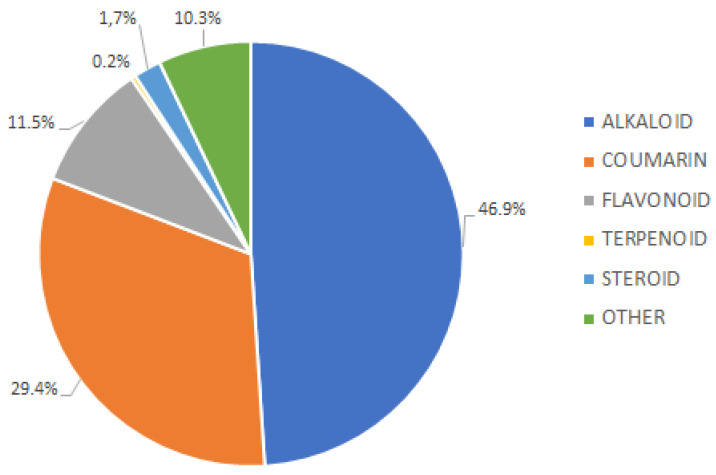
Distribution of compound groups from the Murraya genus.

A total of 413 compounds were isolated based on the data obtained from the literature published between 1965 and 2023. The compounds isolated from the stem bark, bark, roots, leaves, and twigs of *Murraya* species included alkaloids, coumarin, flavonoids, steroids, terpenoids, and other components. Furthermore, previous reports suggested that alkaloids were the dominant metabolites, with a total of 193 compounds (46.9%), followed by coumarin and flavonoid with 121 (29.3%) and 48 (10.3%), respectively (Figure 3). 

### 4.2. Alkaloid

At present, a total of 193 alkaloids have been identified in the form of carbazole, pyridine, pyrrole, *N*-substituted, indole, and dimerics, as shown in Table 1. The pyridinemonocarboxylate-type alkaloid, namely isomurralonginol nicotinate (**1**), was obtained from the leaves and stem of *M. alata* Drake [17]. Wu et al. [18] reported the presence of four new carbazole types, namely murrayamine F (**2**), murrayamines G (**3**), murrayamines H (**4**), and euchrestifoline (**5**), as well as four compounds (**6**–**10**) in *M. euchrestifolia*. Furthermore, other studies isolated new carbazole types from *M. euchrestifolia,* including murrayamine J (**10**), murrayamine K (**11**), murrayamine I (**12**), murrayamine M (**13**), murrayamine N (**14**), murrayamine D (**15**) [19], murrayamine O (**19**), and murrayamine P (**20**) [20], as well as three other alkaloids (**16**–**18**) [19].

**Figure 4:**
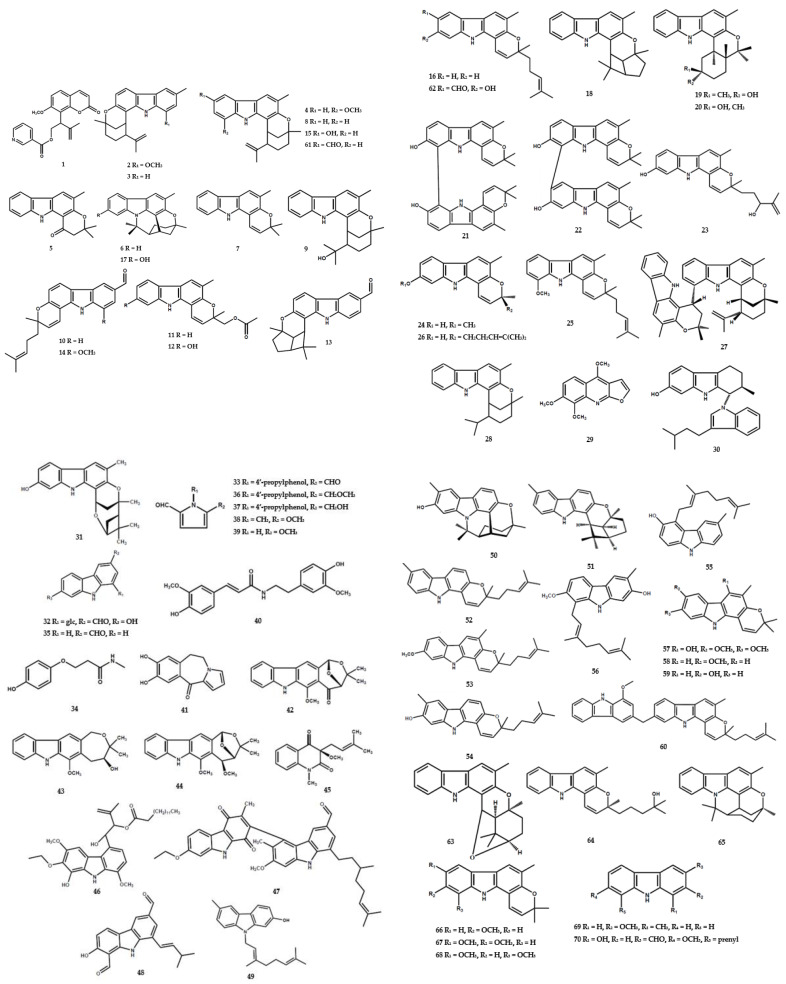
Structures of alkaloids from the *Murraya* genus **1–70**.

The new binary carbazole type, namely bis-7-hydroxygirinimbine A (**21**) and bis-7-hydroxygirinimbine B (**22**), were isolated from the leaves of *M. euchrestifolia* [21]. A total of four carbazole-derivative-type alkaloids (**23**–**26**) were also isolated from *M. euchrestifolia* [19] along with a few other *Murraya* species [2]. McPhail et al. [22] reported the presence of a novel biscarbazole alkaloid, (+)-murrafoline (**27**), from the root bark of *M. euchrestifolia*. Furthermore, methyl-2-methyl-4-(N-2″b-methyl-1″,2″,3″,4″-tetrahydro-carbazol-1″a-ylindol-3′-yl)-butanoate (**30**) was found as a novel indole dimer from the root part of *M. gleinei* [23] (Figure 4).

**Table 1:** Alkaloids from the *Murraya* genus.

Compounds	Part of Plant	Source	References
isomurralonginol nicotinate (**1**)	leaves and stems	*M. alata* Drake	[17]
murrayamines F (**2**)	leaves	*M. euchrestifolia*	[18]
murrayamines G (**3**)	leaves	*M. euchrestifolia*	[18]
murrayamines H (**4**)	leaves	*M. euchrestifolia*	[18]
euchrestifoline (**5**)	leaves	*M. euchrestifolia*	[18]
murrayazoline (**6**)	leaves	*M. euchrestifolia*	[18]
girinimbine (**7**)	leaves	*M. euchrestifolia* *M. koenigii* *M. microphylla*	[18,19,21,24,25] [26,27,28] [2,29]
murrayazolidine (**8**)	leaves	*M. euchrestifolia* *M. koenigii*	[18] [26,30]
murrayazolinine (**9**)	leaves	*M. koenigii * *M. euchrestifolia* *M. koenigii*	[4] [18] [26]
murrayamine-J (**10**)	leaves	*M. euchrestifolia* *M. koenigii* *M. microphylla*	[19] [26] [29]
murrayamine-K (**11**)	leaves	*M. euchrestifolia*	[19]
murrayamine-l (**12**)	leaves	*M. euchrestifolia*	[19]
murrayamine-M (**13**)	leaves	*M. euchrestifolia*	[19]
murrayamine-N (**14**)	leaves	*M. euchrestifolia*	[19]
murrayamine-D (**15**)	leaves	*M. euchrestifolia*	[19]
mahanimbine (**16**)	leaves	*M. euchrestifolia* *M. koenigii* *M. microphylla*	[19,24] [26,27,28,30,31,32] [2,29]
murrayamine-E (**17**)	leaves	*M. euchrestifolia* *M. koenigii*	[19] [31]
bicyclomahanimbine (**18**)	leaves	*M. euchrestifolia* *M. koenigii*	[19] [26,31]
murrayamine-O (**19**)	root bark	*M. euchrestifolia*	[20]
murrayamine-P (**20**)	root bark	*M. euchrestifolia*	[20]
bis-7-hydroxygirinimbine-A (**21**)	leaves	*M. euchrestifolia*	[21]
bis-7-hydroxygirinimbine-B (**22**)	leaves	*M. euchrestifolia*	[21]
murrayamine-C (**23**)	leaves	*M. euchrestifolia* *M. koenigii*	[24,25] [26]
murrayamine-A (**24**)	leaves	*M. euchrestifolia* *M. koenigii* *M. microphylla*	[19,24] [27] [2,29]
murrayamine-B (**25**)	leaves	*M. euchrestifolia*	[24]
mahanine (**26**)	leaves	*M. euchrestifolia* *M. microphylla* *M. koenigii*	[19,24] [2,29] [28,32,33]
(+)-murrafoline (**27**)	root bark	*M. euchrestifolia*	[22]
exozoline (**28**)	stem bark	*M. exotica L.*	[34]
skimianine (**29**)	leaves	*M. gleinei*	[35]
methyl 2-methyl-4-(*N*-2″b-methyl-1″,2″,3″,4″-tetrahydro-carbazol-1″a-ylindol-3′-yl)bustanoate (**30**)	root	*M. gleinei*	[23]
(1′*R*,3′*R*,4′*R*,6′*S*)-endocycliomurrayamine-A (**31**)	whole plant	*M. koenigii*	[4]
3-formyle-7-hydroxy-9H-carbazole-1-*O*-β-D-glucopyranoside (**32**)	whole plant	*M. koenigii*	[4]
4′-hydroxyphenyl-6-ethyl-1H-pyrrole-2-carboxalde-hyde (**33**)	whole plant	*M. koenigii*	[4]
4-hydroxyphenoxy-*N*-methyl-propanamide (**34**)	whole plant	*M. koenigii*	[4]
3-formylcarbazole (**35**)	whole plant	*M. koenigii* *M. kwangsiensis*	[4,28] [3]
pyrrolezanthine-6-methyl ether (**36**)	whole plant	*M. koenigii*	[4]
pyrolezanthine (**37**)	whole plant	*M. koenigii*	[4]
5-hydroxymethyl-1-methylpyrrol-2-carbaldehyde (**38**)	whole plant	*M. koenigii*	[4]
2-formyl-5-hydroxymethyl-pyrrole (**39**)	whole plant	*M. koenigii*	[4]
*N*-trans-feruloyl-3′-*O*-methyldopamine (**40**)	whole plant	*M. koenigii*	[4]
portulacatone (**41**)	whole plant	*M. koenigii*	[4]
claulansium A (**42**)	whole plant	*M. koenigii*	[4]
claulansium B (**43**)	whole plant	*M. koenigii*	[4]
1′-omethylclaulamine B (**44**)	whole plant	*M. koenigii*	[4]
dunnine E (**45**)	whole plant	*M. koenigii*	[4]
mukoenigatin (**46**)	aerial part	*M. koenigii*	[36]
bikoeniquinonine (**47**)	aerial part	*M. koenigii*	[36]
murrayadinal (**48**)	aerial part	*M. koenigii*	[36]
karapinchamines A (**49**)	leaves	*M. koenigii*	[31]
karapinchamines B (**50**)	leaves	*M. koenigii*	[31]
bicyclomahanimbicine (**51**)	leaves	*M. koenigii*	[31]
mahanimbicine (**52**)	leaves	*M. koenigii*	[31]
methylmahanimbine (**53**)	leaves	*M. koenigii*	[31]
pyrayafoline D (**54**)	leaves	*M. koenigii* *M. kwangsiensis*	[31,32] [3]
eustifolin (**55**)	leaves	*M. koenigii*	[31]
euchrestine-B (**56**)	leaves	*M. koenigii*	[31,32]
kurryam (**57**)	seeds	*M. koenigii*	[5]
koenimbine (**58**)	seeds	*M. koenigii* *M. microphylla*	[5,26,27,32] [2,29]
koenine (**59**)	seeds	*M. koenigii* *M. microphylla*	[5,27,32] [2]
murrayakonine A (**60**)	stems and leaves	*M. koenigii*	[26]
murrayakonine B (**61**)	stems and leaves	*M. koenigii*	[26]
murrayakonine C (**62**)	stems and leaves	*M. koenigii*	[26]
murrayakonine D (**63**)	stems and leaves	*M. koenigii*	[26]
mahanimbinine (**64**)	stems and leaves	*M. koenigii* *M. microphylla*	[26] [29]
currayangine (**65**)	stems and leaves	*M. koenigii*	[26]
*O*-methylmurrayamine-A (**66**)	stems and leaves	*M. koenigii* *M. microphylla*	[26] [2]
koenigicine (**67**)	stems and leaves	*M. koenigii*	[26]
mukonicine (**68**)	stems and leaves	*M. koenigii*	[26]
2-methoxy-3-methyl-9H-carbazole (**69**)	stems and leaves	*M. koenigii*	[26]
1-hydroxy-7-methoxy-8-(3-methylbut-2-en-1-yl)-9H-carbazole-3-carbaldehyde (**70**)	stems and leaves	*M. koenigii*	[26]
8,8″-biskoenigine (**71**)	stems and leaves	*M. koenigii*	[26,27]
clauraila A (**72**)	stems and leaves	*M. koenigii*	[26]
*N*-benzyl carbazole-A (**73**)	whole plant	*M. koenigii*	[27]
*N*-benzyl carbazole-B (**74**)	whole plant	*M. koenigii*	[27]
isokoenidine (**75**)	whole plant	*M. koenigii* *M. microphylla*	[27] [2]
iso-koenigine (**76**)	whole plant	*M. koenigii*	[27]
koenigine (**77**)	whole plant	*M. koenigii* *M. microphylla*	[27,32] [2,29]
koenidine (**78**)	whole plant	*M. koenigii* *M. microphylla*	[27,32] [2,29]
murrayakoeninol (**79**)	leaves	*M. koenigii*	[37]
koenoline (**80**)	whole plant	*M. koenigii*	[27,38]
*N*-methoxy-3-hydroxymethyl-9H-carbazole (**81**)	whole plant	*M. koenigii*	[27]
3-hydroxymethyl-9-H-carbazole (**82**)	whole plant	*M. koenigii*	[27]
*O*-demethylmurrayanine (**83**)	whole plant	*M. koenigii* *M. kwangsiensis*	[27] [3]
murrastanine A (**84**)	bark and leaves	*M. koenigii*	[39]
murrastinine A (**85**)	bark and leaves	*M. koenigii*	[39]
murrastinine B (**86**)	bark and leaves	*M. koenigii*	[39]
murrastinine C (**87**)	bark and leaves	*M. koenigii* *M. microphylla*	[39] [2,29]
murrayatanine-A (**88**)	bark and leaves	*M. koenigii*	[39]
bismahanimboline (**89**)	bark and leaves	*M. koenigii*	[39]
murrafoline-I (**90**)	leaves	*M. koenigii*	[32]
mahabinine-A (**91**)	leaves	*M. koenigii*	[32]
bisgerayafoline D (**92**)	fruit	*M. koenigii*	[33]
bismahanimbinol (**93**)	fruit	*M. koenigii*	[33]
bispyrayafoline (**94**)	fruit	*M. koenigii*	[33]
*O*-methyl mahanine (**95**)	fruit	*M. koenigii*	[33]
*O*-methyl mukonal (**96**)	fruit	*M. koenigii*	[33]
3,3′-[oxybis(methylene)]bis(9-methoxy-9H-carbazole) (**97**)	stem bark	*M. koenigii*	[28]
murrayanine (**98**)	stem bark	*M. koenigii* *M. kwangsiensis* *M. microphylla*	[7,26,27,28,38] [3] [29]
3-formyl-9-methoxycarbazole (**99**)	stem bark	*M. koenigii*	[28]
carbazole-3-carboxylic acid (**100**)	stem bark	*M. koenigii*	[28]
koenigine-quinone A (**101**)	stem bark	*M. koenigii*	[40]
koenigine-quinone B (**102**)	stem bark	*M. koenigii*	[40]
bismurrayafoline D (**103**)	leaves	*M. euchrestifolia*	[41]
bismurrayafoline E (**104**)	leaves	*M. koenigii*	[42]
9-carbethoxy-3-methylcarbazole (**105**)	roots	*M. koenigii*	[43]
9-formyl-3-methylcarbazole (**106**)	roots	*M. koenigii*	[43]
3-methyl-carbazole (**107**)	roots	*M. koenigii*	[43]
isomahanine (**108**)	fruits	*M. koenigii* *M. euchrestifolia*	[30] [19]
murrayanol (**109**)	fruits	*M. koenigii*	[30]
mukonal (**110**)	whole plant	*M. koenigii*	[44]
mukonicine (**111**)	leaves	*M. koenigii*	[45]
isomurrayazoline (**112**)	stem bark	*M. koenigii*	[46]
mukonine (**113**)	root	*M. koenigii*	[47]
(−)-bispyrayafoline C (**114**)	leaves and stems	*M. kwangsiensis*	[3]
(+)-bispyrayafoline C (**115**)	leaves and stems	*M. kwangsiensis*	[3]
(−) kwangsine A (**116**)	leaves and stems	*M. kwangsiensis*	[3]
(−) kwangsine A (**117**)	leaves and stems	*M. kwangsiensis*	[3]
(−) kwangsine B (**118**)	leaves and stems	*M. kwangsiensis*	[3]
(+) kwangsine B (**119**)	leaves and stems	*M. kwangsiensis*	[3]
(−) kwangsine C (**120**)	leaves and stems	*M. kwangsiensis*	[3]
(+) kwangsine C (**121**)	leaves and stems	*M. kwangsiensis*	[3]
kwangsine D (**122**)	leaves and stems	*M. kwangsiensis*	[3]
kwangsine E (**123**)	leaves and stems	*M. kwangsiensis*	[3]
kwangsine F (**124**)	leaves and stems	*M. kwangsiensis*	[3]
kwangsine G (**125**)	leaves and stems	*M. kwangsiensis*	[3]
kwangsine H (**126**)	leaves and stems	*M. kwangsiensis*	[3]
kwangsine I (**127**)	leaves and stems	*M. kwangsiensis*	[3]
kwangsine J (**128**)	leaves and stems	*M. kwangsiensis*	[3]
kwangsine K (**129**)	leaves and stems	*M. kwangsiensis*	[3]
kwangsine L (**130**)	leaves and stems	*M. kwangsiensis*	[3]
kwangsine M (**131**)	leaves and stems	*M. kwangsiensis*	[3]
pyrayaquinone B (**132**)	leaves and stems	*M. kwangsiensis*	[3]
pyrayafoline C (**133**)	leaves and stems	*M. kwangsiensis* *M. microphylla*	[3] [2]
euchrestine-A (**134**)	leaves and stems	*M. kwangsiensis*	[3]
euchrestine-C (**135**)	leaves and stems	*M. kwangsiensis*	[3]
2-hydroxy-3-methylcarbazole (**136**)	leaves and stems	*M. kwangsiensis* *M. microphylla*	[3] [29]
1-hydroxy-3-methyl-9H-carbazole (**137**)	leaves and stems	*M. kwangsiensis*	[3]
3-hydro-xymethyl-9H-carbazole (**138**)	leaves and stems	*M. kwangsiensis*	[3]
3-(methoxymethyl)carbazole (**139**)	leaves and stems	*M. kwangsiensis*	[3]
1-methoxy-3-(methoxymethyl)carbazole (**140**)	leaves and stems	*M. kwangsiensis*	[3]
claulansine Q (**141**)	leaves and stems	*M. kwangsiensis*	[3]
claulansine R (**142**)	leaves and stems	*M. kwangsiensis*	[3]
3-carboxylic acid carbazole (**143**)	leaves and stems	*M. kwangsiensis*	[3]
clausine E (**144**)	leaves and stems	*M. kwangsiensis*	[3]
3-methyl-9H-carbazole (**145**)	leaves and stems	*M. kwangsiensis*	[3]
murrayafoline A (**146**)	leaves and stems	*M. kwangsiensis*	[3]
(+)-microphylines N (**147**)	leaves and stems	*M. microphylla*	[2]
(–)-microphylines N (**148**)	leaves and stems	*M. microphylla*	[2]
(+)-microphylines O (**149**)	leaves and stems	*M. microphylla*	[2]
(–)-microphylines O (**150**)	leaves and stems	*M. microphylla*	[2]
(+)-microphylines P (**151**)	leaves and stems	*M. microphylla*	[2]
(–)-microphylines P (**152**)	leaves and stems	*M. microphylla*	[2]
(+)-microphylines Q (**153**)	leaves and stems	*M. microphylla*	[2]
(–)-microphylines Q (**154**)	leaves and stems	*M. microphylla*	[2]
(+)-microphylines R (**155**)	leaves and stems	*M. microphylla*	[2]
(**–**)-microphylines R (**156**)	leaves and stems	*M. microphylla*	[2]
isogirinimbine (**157**)	leaves and stems	*M. microphylla*	[2]
heptazolidine (**158**)	leaves and stems	*M. microphylla*	[2]
(−)-mahanimbicine (**159**)	leaves and stems	*M. microphylla*	[2]
(−)-pyrayafoline D (**160**)	leaves and stems	*M. microphylla*	[2]
O-(−)-methylpyrayafoline D (**161**)	leaves and stems	*M. microphylla*	[2]
(−)-murrayamine-J (**162**)	leaves and stems	*M. microphylla*	[2]
(−)-murrayamine-B (**163**)	leaves and stems	*M. microphylla*	[2]
(−)-6-hydroxymahanimbine (**164**)	leaves and stems	*M. microphylla*	[2]
(2′*S*,3′*R*)-microphyline K (**165**)	leaves and stems	*M. microphylla*	[29]
(2′*R*,3′*S*)-microphyline K (**166**)	leaves and stems	*M. microphylla*	[29]
microphyline L (**167**)	leaves and stems	*M. microphylla*	[29]
microphyline M (**168**)	leaves and stems	*M. microphylla*	[29]
6-hydroxygirinimbine (**169**)	leaves and stems	*M. microphylla*	[29]
3-formyl-1-hydroxycarbazole (**170**)	leaves and stems	*M. microphylla*	[29]
clausine P (**171**)	leaves and stems	*M. microphylla*	[29]
9H-1-hydroxy-7-methoxy-8-(3-methyl-2-buten-1-yl)-carbazole-3-carboxaldehyde (**172**)	leaves and stems	*M. microphylla*	[29]
clausine Q (**173**)	leaves and stems	*M. microphylla*	[29]
1-hydroxy-3-methylcarbazole (**174**)	leaves and stems	*M. microphylla*	[29]
carbalexin B (**175**)	leaves and stems	*M. microphylla*	[29]
murrayacarine (**176**)	root bark	*M. omphalocarpa*	[48]
3-formylindole (**177**)	stem bark	*M. omphalocarpa*	[49]
paniculidines D (**178**)	roots	*M. paniculata*	[50]
paniculidines E (**179**)	roots	*M. paniculata*	[50]
paniculidines F (**180**)	roots	*M. paniculata*	[50]
paniculidines A (**181**)	roots	*M. paniculata*	[50,51]
paniculidines B (**182**)	roots	*M. paniculata*	[50,51]
paniculidines C (**183**)	roots	*M. paniculata*	[50,51]
tanakine (**184**)	roots	*M. paniculata*	[50]
indol-3-carbaldehyde (**185**)	roots	*M. paniculata*	[50]
yuehchukene (**186**)	roots	*M. paniculata*	[8,50]
alanditrypinone (**187**)	leaves	*M. paniculata*	[52]
alantryphenone (**188**)	leaves	*M. paniculata*	[52]
alantrypinene (**189**)	leaves	*M. paniculata*	[52]
alantryleunone (**190**)	leaves	*M. paniculata*	[52]
murrayaculatine (**191**)	flower	*M. paniculata*	[53]
murradiate (**192**)	leaves and stems	*M. tetramera*	[54]
murradiol (**193**)	leaves and stems	*M. tetramera*	[54]

A further investigation of *M. koenigii* by Wei et al. [4] identified three new alkaloid derivatives, including two carbazoles, namely (1′*R*,3′*R*,4′*R*,6′*S*)-endocycliomurrayamine-A (**31**), 3-formyle-7-hydroxy-9H-carbazole-1-*O*-β-D-glucopyranoside (**32**), and a pyrole-type 4′-hydroxyphenyl-6-ethyl-1H-pyrrole-2-carboxaldehyde (**33**). The aliphatic alkaloid-type (**34,40**), carbazole-type (**35**), and three substituted pyrole-type (**36**–**39**) compounds were also identified in the species [4]. 

**Figure 5:**
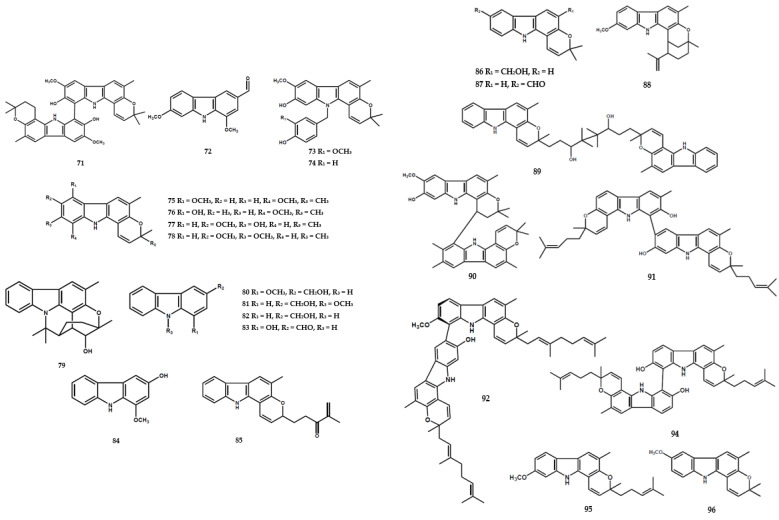

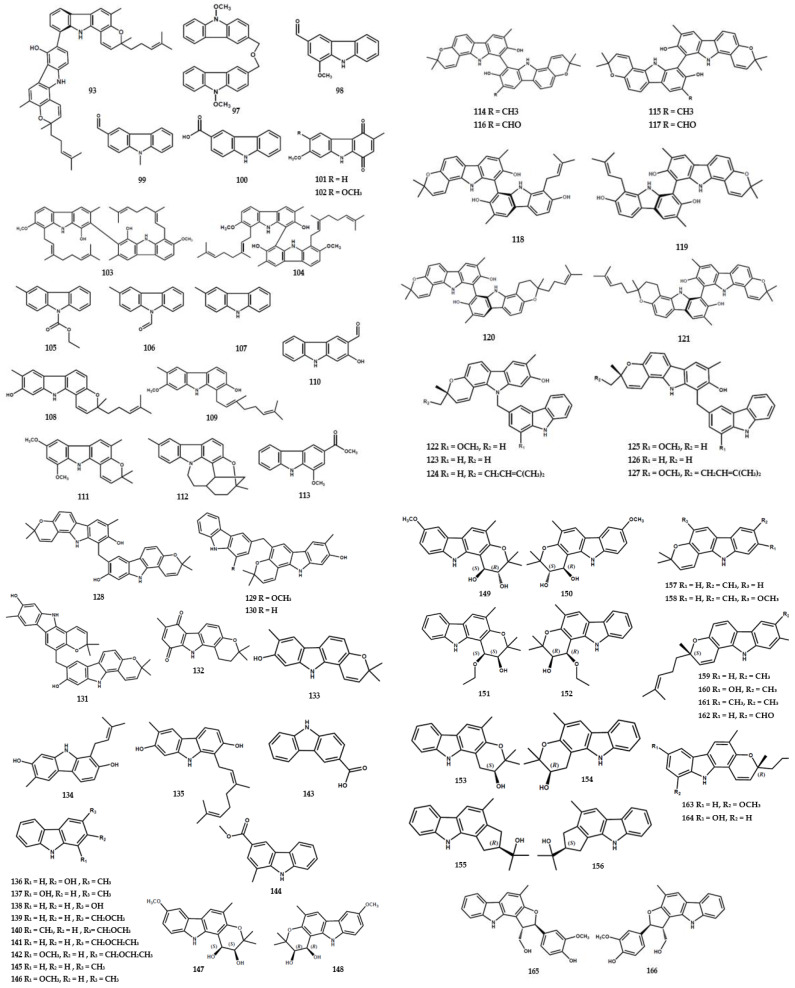

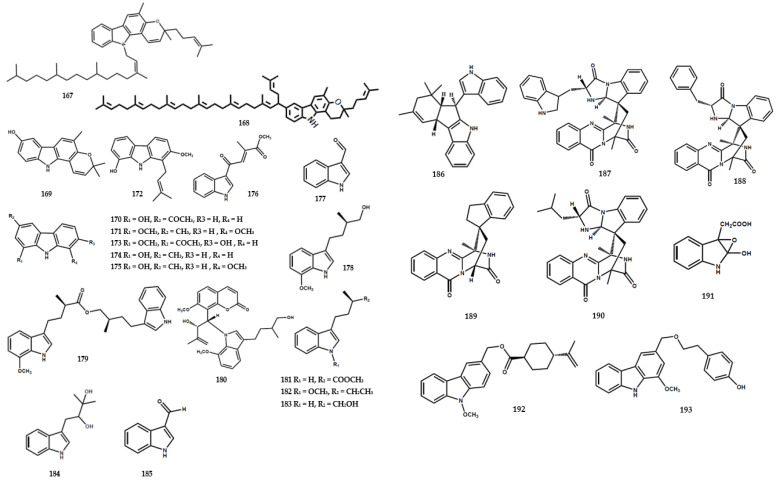
Structures of alkaloids from the *Murraya* genus **71–193**.

A previous study reported the isolation of the lactam derivatives, portulacatone (**41**), along with sixteen other alkaloids, from *M. koenigii* [4]. A total of three oxepane-carbazole derivatives, namely claulansium A (**42**), claulansium B (**43**), and 1′-omethylclaulamine B (**44**), and one other compound (**45**) were also identified [4]. Furthermore, the aerial parts of *M. koenigii* contained the dimer alkaloid, bikoeniquinonine (**47**) [36], along with two dimer types, namely murrayakonine A (**60**) [26] and 8,8′-biskoenigine (**71**) [27] (Figure 5).

In further phytochemical studies, substituted carbazole and *N*-substituted carbazole structures were commonly found in the *Murraya* genus. Naz et al. [36] reported the isolation of mukoenigatin (**46**) and murrayadinal (**48**) from the aerial parts of *M. koenigii*. Karapinchamines A (**49**), karapinchamines B (**50**), eustifolin (**55**), and euchrestine B (**56**) were also obtained from its leaves [31]. An investigation by Nalli et al. [26] identified three *N*-substituted carbazoles, including 2-methoxy-3-methyl-9H-carbazole (**69**), 1-hydroxy-7-methoxy-8-(3-methylbut-2-en-1-yl)-9H-carbazole-3-carbaldehyde (**70**), and clauraila A (**72**).

The benzo[a]carbazole-type alkaloids were frequently found in the *Murraya* genus. Nakamura et al. [31] identified the presence of mahanimbicine (**52**) and two other compounds (**53–54**) in the leaves of *M. koenigii*. Another three compounds, namely kurryam (**57**), koenimbine (**58**), and koenine (**59**), were also obtained from its seeds [5]. Furthermore, *O*-methylmurrayamine-A (**66**), koenigicine (**67**), mukonicine (**68** [26], *N*-benzyl carbazole-A (**73**), *N*-benzyl carbazole-B (**74**) [27], and murrastinine A-C (**85–87**) [39] were identified from *M. koenigii* and several other species.

Alkaloids were also identified in other forms, including substituted indole derivative types, encompassing 3-formylindole (**177**) from *M. omphalocarpa* [49], paniculidines D (**178**), paniculidines E (**179**), and seven compounds (**180**–**186**) from *M. paniculata* [50]. 

### 4.3. Coumarins

Several coumarins were identified in *Murrya* species, such as *M. alata*, *M. gleinei*, *M. paniculata,* and *M. exotica*. At present, a total of 121 compounds in this category have been reported, as shown in Table 2. Furthermore, these compounds were identified in the form of substituted simple coumarin, coumarin glycoside, alkoxycoumarin, 8-alkyl substituted, and furano type. Methoxy-substituted analog type, namely muralatin R (**194**), was obtained from *M. alata* [55]. Several coumarins have also been isolated from the same species, including meranzin (**195**), phebalosin (**196**), muralatin N (**203**), and meranzin hydrate (**208**). A previous study reported the presence of coumarin glycoside type, namely muralatin Q (**213**), in *M. alata* [17]. 

**Figure 6:**
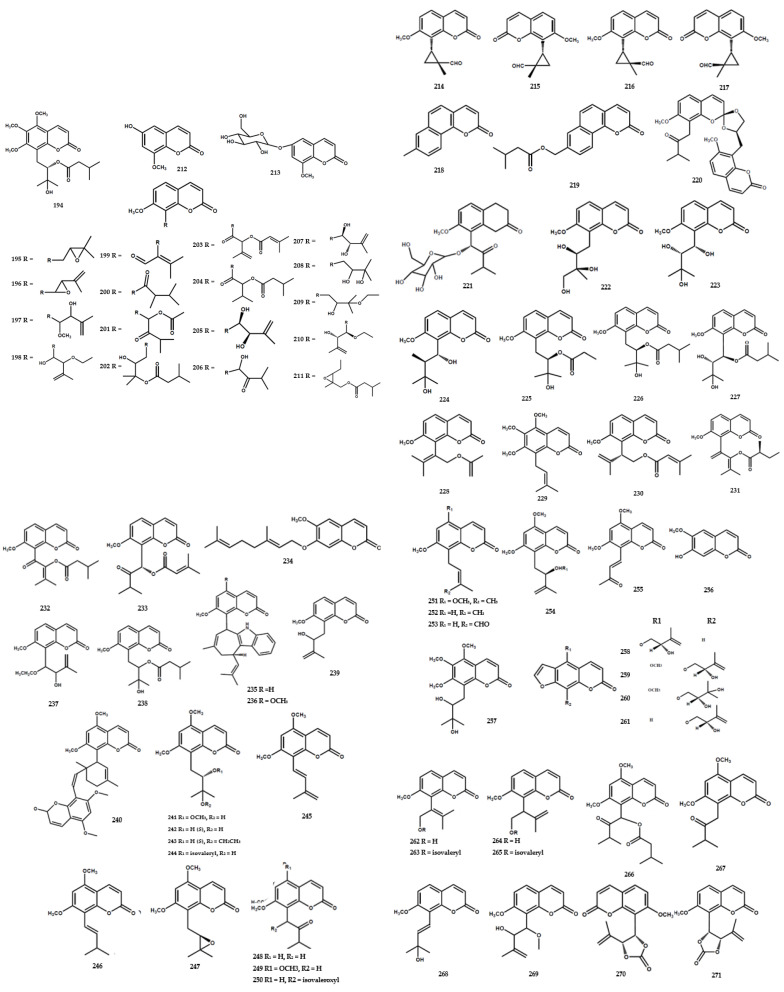

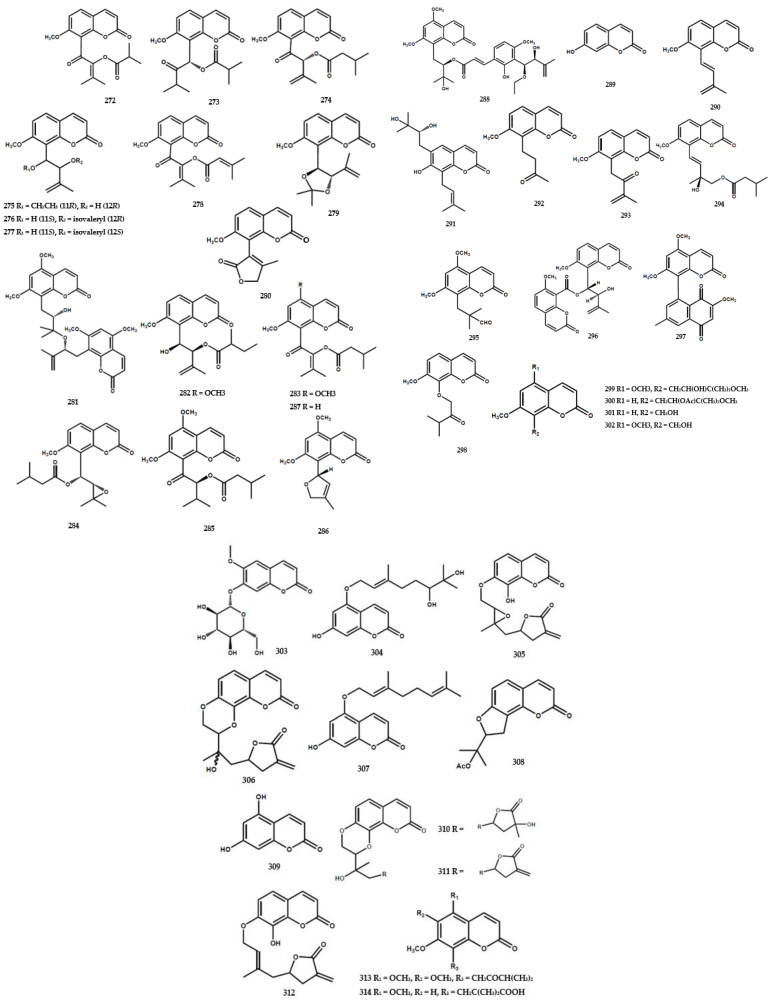
Structures of coumarins from the *Murraya* genus **194–314**.

Coumarin-substituted cyclopropane was isolated from *M. exotica* in the form of an enantiomer*,* muratin A (**214–215**), and muratin B (**216–217**) [56]. Another study reported the isolation of a glycoside coumarin derivative, muratin F (**221**), from the same species [56] (Figure 6).

**Table 2:** Coumarins from the *Murraya genus*.

Compounds	Part of Plant	Source	References
muralatin R (**194**)	leaves	*M. alata* Drake	[55]
meranzin (**195**)	leaves and stems	*M. alata* Drake	[17]
phebalosin (**196**)	leaves and stems	*M. alata* Drake	[17]
murracarpin (**197**)	leaves and stems	*M. alata* Drake	[17]
2′-*O*-ethylmurrangatin (**198**)	leaves and stems	*M. alata* Drake *M. paniculata*	[17] [57]
muralongin (**199**)	leaves and stems	*M. alata* Drake	[17]
muralatin L (**200**)	leaves and stems	*M. alata* Drake	[17]
hainanmurpanin (**201**)	leaves and stems	*M. alata* Drake *M. gleinei*	[17] [35]
muralatin M (**202**)	leaves and stems	*M. alata* Drake *M. gleinei*	[17] [35,58]
muralatin N (**203**)	leaves and stems	*M. alata* Drake	[17]
muralatin O (**204**)	leaves and stems	*M. alata* Drake *M. paniculata*	[17] [57,59]
murangatin (**205**)	leaves and stems	*M. alata* Drake *M. gleinei* *M. paniculata* *M. elongata* *M. omphalocarpa*	[17] [35,58] [51,57] [60] [61]
murpaniculol (**206**)	leaves and stems	*M. alata* Drake	[17]
minumicrolin (**207**)	leaves and stems	*M. alata* Drake *M. gleinei* *M. omphalocarpa* *M. paniculata * *M. elongata* *M. exotica*	[17] [35,58] [48,61] [57,59] [62] [63]
meranzin hydrate (**208**)	leaves and stems	*M. alata* Drake	[17]
yuehgesin-C (**209**)	leaves and stems	*M. alata* Drake *M. omphalocarpa* *M. paniculata*	[17] [61] [57,59]
muralatin K (**210**)	leaves and stems	*M. alata* Drake *M. gleinei * *M. exotica*	[17] [35] [63]
muralatin P (**211**)	leaves and stems	*M. alata* Drake	[17]
muralatin K (**212**)	leaves and stems	*M. alata* Drake *M. paniculata*	[17] [59]
muralatin Q (**213**)	leaves and stems	*M. alata Drake*	[17]
(−) murratin A (**214**)	leaves and twigs	*M. exotica L.*	[56]
(+) murratin A (**215**)	leaves and twigs		
(−) murratin B (**216**)	leaves and twigs	*M. exotica L.*	[56]
(+) murratin B (**217**)	leaves and twigs		
murratin C (**218**)	leaves and twigs	*M. exotica L.*	[56]
murratin D (**219**)	leaves and twigs	*M. exotica L.*	[56]
murratin E (**220**)	leaves and twigs	*M. exotica L.*	[56]
murratin F (**221**)	leaves and twigs	*M. exotica L.*	[56]
murratin G (**222**)	leaves and twigs	*M. exotica L.*	[56]
murratin H (**223**)	leaves and twigs	*M. exotica L.*	[56]
murratin I (**224**)	leaves and twigs	*M. exotica L.*	[56]
murratin J (**225**)	leaves and twigs	*M. exotica L.*	[56]
murratin K (**226**)	leaves and twigs	*M. exotica L.*	[56]
murratin L (**227**)	leaves and twigs	*M. exotica L.*	[56]
murratin M (**228**)	leaves and twigs	*M. exotica L.*	[56]
muralatin C (**229**)	leaves and twigs	*M. exotica L.*	[56]
2-(7-methoxy-2-oxochromen-8-yl)-3-methylbut-2-enyl] 3-methylbut-2-enoate (**230**)	leaves and twigs	*M. exotica L.*	[56]
panitin C (**231**)	leaves and twigs	*M. paniculata * *M. exotica L.*	[57,59] [56]
exotimarin H (**232**)	leaves and twigs leaves and stems	*M. exotica L. * *M. paniculata*	[56] [59]
epimurpaniculol senecioate (**233**)	leaves and twigs	*M. exotica L.*	[56]
7-geranyloxy-6-methoxycoumarin (**234**)	leaves and twigs	*M. paniculata* *M. exotica L.*	[57] [56]
exotines A (**235**)	roots	*M. exotica L.*	[64]
exotines B (**236**)	roots	*M. exotica L.*	[64]
murraxocin (**237**)	roots	*M. exotica L.*	[65]
murrayatin (**238**)	leaves	*M. exotica L. * *M. paniculata*	[66] [67]
auraptenol (**239**)	leaves	*M. exotica L.*	[63]
mexolide (**240**)	stem bark	*M. exotica L.*	[68]
murraglenin (**241**)	leaves	*M. omphalocarpa*	[48]
mexoticin (**242**)	roots	*M. gleinei * *M. omphalocarpa* *M. paniculata*	[35,58] [48,69] [57,70]
5,7-dimethoxy-8-(2-hydroxyl-3-ethoxy-3-methylbutyl coumarin (**243**)	roots	*M. paniculata*	[57]
5-methoxymurrayatin (**244**)	roots	*M. paniculata*	[57,59]
gleinadiene (**245**)	roots	*M. gleinei*	[58]
gleinene (**246**)	roots	*M. gleinei*	[58]
sibiricin (**247**)	roots	*M. gleinei* *M. paniculata*	[35] [57]
isomeranzin (**248**)	roots	*M. paniculata*	[57]
murrayone (**249**)	roots	*M. paniculata*	[57]
paniculatin (**250**)	roots	*M. paniculata*	[57,59]
coumurrayin (**251**)	roots	*M. paniculata* *M. omphalocarpa*	[57,70,71] [48,69]
osthol (**252**)	roots	*M. paniculata*	[51,57]
7-methoxy-8-(3′-formylbut-2′-enyl)-coumarin (**253**)	roots	*M. paniculata*	[57]
omphamurin (**254**)	leaves	*M. omphalocarpa* *M. paniculata*	[48] [57]
toddalenone (**255**)	roots	*M. gleinei* *M. omphalocarpa*	[58] [61]
scopoletin (**256**)	leaves	*M. gleinei*	[35]
murragleinin (**257**)	leaves	*M. gleinei* *M. paniculata*	[35] [70]
gosferol (**258**)	seeds	*M. koenigii*	[72]
neobyakangelicol (**259**)	seeds	*M. koenigii*	[72]
byakangelicin (**260**)	seeds	*M. koenigii*	[72]
isogosferol (**261**)	seeds	*M. koenigii*	[72]
murralonginol (**262**)	roots	*M. paniculata*	[57]
murralonginol isovalerate (**263**)	roots	*M. paniculata*	[57]
isomurralonginol (**264**)	roots	*M. paniculata*	[57]
isomurralonginol isovalerate (**265**)	roots	*M. paniculata* *M. omphalocarpa*	[57] [61]
omphamurrayone (**266**)	leaves	*M. omphalocarpa*	[61]
5,7-dimethoxy-8-(3′-methyl-2′-oxobutyl) coumarin (**267**)	root bark	*M. omphalocarpa* *M. paniculata*	[48,69] [70,73]
murraol (**268**)	root bark	*M. omphalocarpa*	[48]
(+)-murracarpin (**269**)	root bark	*M. omphalocarpa* *M. paniculata*	[48] [70]
(+)-murpanitin A (**270**)	leaves and stems	*M. paniculata*	[59]
(−)-murpanitin A (**271**)	leaves and stems	*M. paniculata*	[59]
murpanitins B (**272**)	leaves and stems	*M. paniculata*	[59]
murpanitins C (**273**)	leaves and stems	*M. paniculata*	[59]
murpanitins D (**274**)	leaves and stems	*M. paniculata*	[59]
murpanicin (**275**)	leaves and stems	*M. paniculata*	[57,59]
minumicrolin isovalerate (**276**)	leaves and stems	*M. paniculata*	[59]
murrangatin isovalerate (**277**)	leaves and stems	*M. paniculata*	[57,59]
kimcuongin (**278**)	leaves and stems	*M. paniculata*	[59]
minumicrolin acetonide (**279**)	leaves and stems	*M. paniculata*	[57,59]
microminutin (**280**)	leaves and stems	*M. paniculata*	[59]
panitin A (**281**)	roots	*M. paniculata*	[57]
panitin B (**282**)	roots	*M. paniculata*	[57]
panitin D (**283**)	roots	*M. paniculata*	[57]
panitin E (**284**)	roots	*M. paniculata*	[57]
panitin F (**285**)	roots	*M. paniculata*	[57]
panitin G (**286**)	roots	*M. paniculata*	[57]
exotimarin I (**287**)	roots	*M. paniculata*	[57]
10′-ethoxyexotimarin F (**288**)	roots	*M. paniculata*	[57]
Umbelliferone (**289**)	roots	*M. paniculata*	[57] [74]
*trans*-dehydroosthol (**290**)	roots	*M. paniculata*	[57]
6-(2′,3′-dihydroxy-3-methylbutyl)-8-prenylumbelliferone (**291**)	roots	*M. paniculata*	[57]
hassanon (**292**)	roots	*M. paniculata*	[57]
5,7-dimethoxy-8-(3-methyl-2-keto-butyl)coumarin (**293**)	roots	*M. paniculata*	[57]
casegravol isovalerate (**294**)	roots	*M. paniculata*	[57]
seselinal (**295**)	roots	*M. paniculata*	[57]
cladimarin B (**296**)	roots	*M. paniculata*	[57]
toddacoumaquinone (**297**)	roots	*M. paniculata*	[57]
8-(2′,-oxo-3′-methyl)butoxy-7-methoxycoumarin (**298**)	leaves	*M. paniculata*	[67]
omphalocarpin (**299**)	flowers	*M. omphlocarpa* *M. paniculata*	[48] [70]
(−)-murracarpin (**300**)	flowers	*M. omphalocarpa* *M. paniculata*	[48] [70]
murrayacarpin-A (**301**)	flowers	*M. paniculata*	[70]
murrayacarpin-B (**302**)	flowers	*M. paniculata*	[70]
scopolin (**303**)	flowers	*M. paniculata*	[70]
murrayacoumarin A (**304**)	leaves	*M. siamensis*	[74]
murrayacoumarin B (**305**)	leaves	*M. siamensis*	[74]
murrayacoumarin C (**306**)	leaves	*M. siamensis*	[74]
5-geranyloxy-7-hydroxy-coumarin (**307**)	leaves	*M. siamensis*	[74]
columbianetin acetate (**308**)	leaves	*M. siamensis*	[74]
5,7-dihydroxycoumarin (**309**)	leaves	*M. siamensis*	[74]
clauslactone B (**310**)	leaves	*M. siamensis*	[74]
clauslactone A (**311**)	leaves	*M. siamensis*	[74]
clauslactone E (**312**)	leaves	*M. siamensis*	[74]
murrayanone (**313**)	leaves	*M. paniculata*	[73]
murraculatin (**314**)	leaves	*M. paniculata*	[73]

Several studies reported the isolation of various C-8-subtituted coumarins from the *Murraya* genus. Liang et al. [56] reported the presence of murratin G (**222**), murratin H (**223**), murratin I (**224**), murratin J (**225**), murratin K (**226**), murratin L (**227**), murratin M (**228**), muralatin C (**229**), 2-(7-methoxy-2-oxochromen-8-yl)-3-methylbut-2-enyl]-3-methylbut-2-enoate (**230**), and two other compounds (**231** and **233**) from *M. exotica*. Furthermore, exotimarin H (**232**) and 7-geranyloxy-6-methoxycoumarin (**234**) were identified in *M. exotica* [56] and *M. paniculata* [57].

A dimeric coumarin, mexolide (**240**), was identified from the stem bark of *M. exotica* [68]. A previous study reported the presence of a furanocoumarin type, consisting of gosferol (**258**), neobyakangelicol (**259**), byakangelicin (**260**), and isogosferol (**261**), in the seeds of *M. koenigii* [72] (Figure 6). 

### 4.4. Flavonoid

A total of forty-eight flavonoids had been identified in the form of flavone, flavanone, flavanonol, and flavanoid glycoside, as shown in Table 3. A previous study reported the presence of 3,3′,4′,5,5′,7,8-heptamethoxyflavone (**318**) in *M. exotica* in 1970 [75] and further reports isolated 3,5,6,8,3′,4′,5′-heptamethoxyflavone (**317**) from the same species [63]. Furthermore, five flavone types were isolated from *M. paniculata,* encompassing 3′-hydroxy-5,6,7,4′,5′-pentamethoxyflavone (**321**), 5,3′-dihydroxy-6,7,4′,5′-tetramethoxy-flavone (**322**), 5,3′-dihydroxy-7,4′,5′-trimethoxyflavone (**323**), 5-hydroxy-7,8,3′,4′-tetramethoxyflavone (**324**), and 4′-hydroxy-5,6,7,3′,5′-pentamethoxyflavone (**325**) [76].

**Figure 7:**
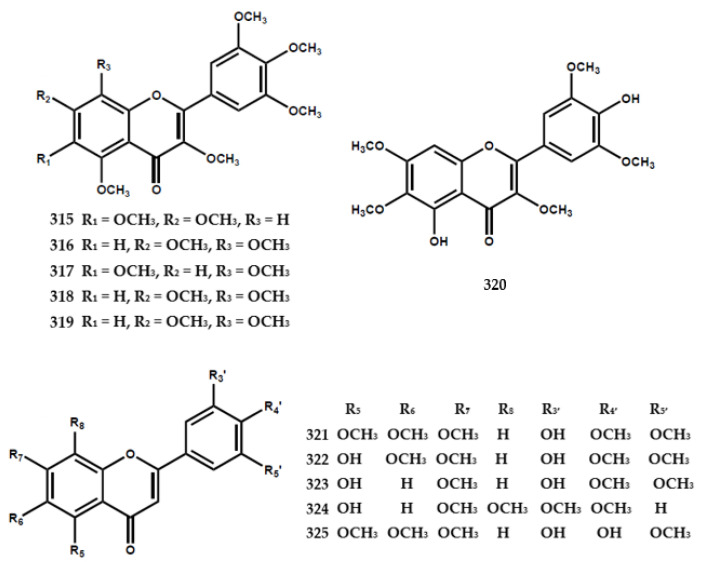

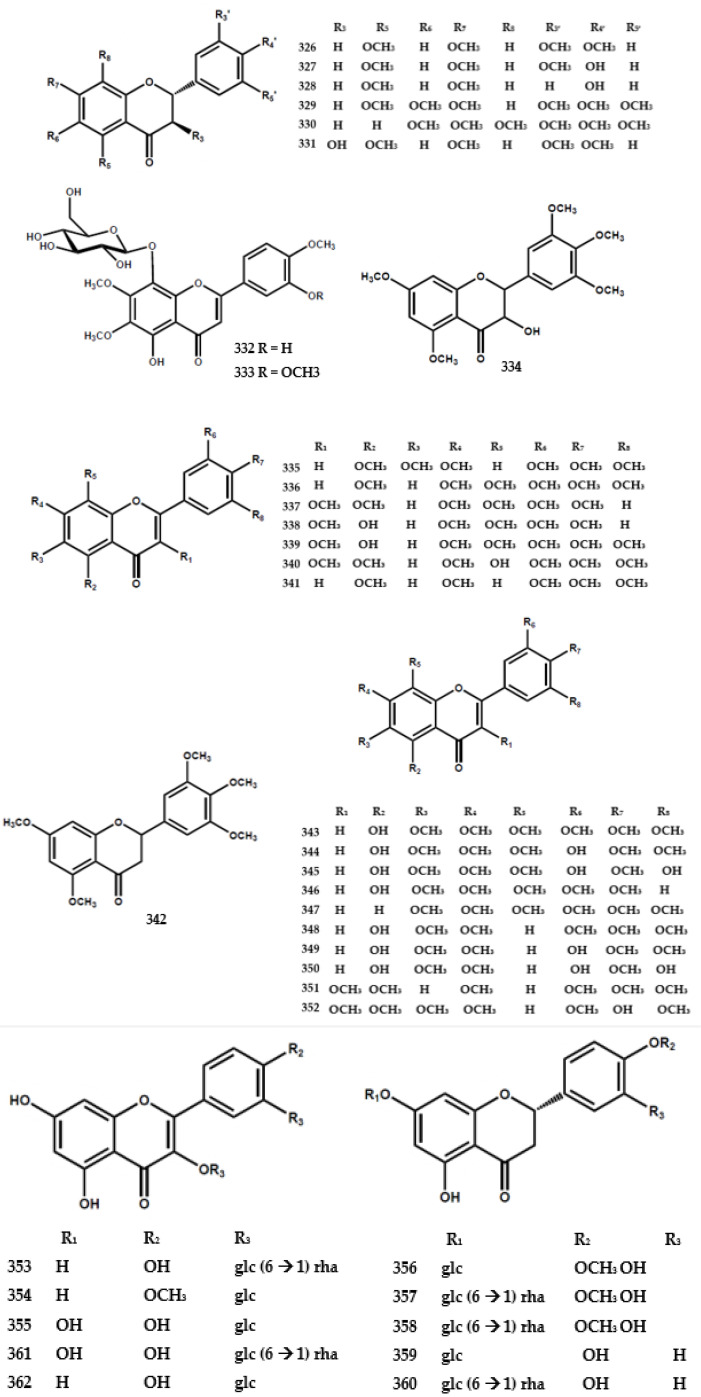
Structures of flavonoids from the *Murraya* genus **315–362**.

Six flavanone types were also identified in *M. paniculata*, including 5,7,3′,4′-tetramethoxyflavanone (**326**), 4′-hydroxy-5,7,3′-trimethoxyflavanone (**327**), 4′-hydroxy-5,7-dimethoxyflavanone (**328**), 5,6,7,3′,4′,5′-hexamethoxyflavanone (**329**), 6,7,8,3′,4′,5′-hexamethoxyflavanone (**330**), and 3-hydroxy-5,7,3′,4′-tetramethoxyflavanone (**331**) [76] (Figure 7). 

The flavanonol-type flavonoid was found in *M. paniculata* and identified as 5,7,3′,4′,5′-pentamethoxyflavanonol (**334**) [77]. Ferracin et al. [77] isolated six flavone types, including 5,6,7,3′,4′,5′-hexamethoxyflavone (**335**), 5,7,8,3′,4′,5′-hexamethoxy-flavone (**336**), 3,5,7,8,-3′,4′-hexamethoxyflavone (**337**), 5-hydroxy-3,7,8,3′,4′-penta-methoxy-flavone (**338**), 5-hydroxy-3,7,8,3′,4′,5′-hexamethoxyflavone (**339**), and 8-hydroxy-3,5,7,3′,4′,5′-hexa-methoxyflavone (**340**) (Figure 7).

**Table 3:** Flavonoids from the *Murraya genus*.

Compounds	Part of Plant	Source	References
3,5,6,7,3′,4′,5′-heptamethoxyflavone (**315**)	leaves and stems	*M. alata* Drake	[17,63]
3,5,7,8,3′,4′,5′-heptamethoxyflavone (**316**)	leaves and stems	*M. alata* Drake	[17]
3,5,6,8,3′,4′,5′-heptamethoxyflavone (**317**)	leaves	*M. exotica L.*	[63]
3,3′,4′,5,5′,7,8-Heptamethoxyflavone (**318**)	leaves	*M. exotica L.*	[75]
Exoticin (**319**)	leaves	*M. gleinei*	[35]
5,4′-dihydroxy-3,6,7,3′,5′-pentamethoxyflavone (**320**)	fresh fruits	*M. omphalocarpa*	[78]
3′-hydroxy-5,6,7,4′,5′-pentamethoxyflavone (**321**)	leaves and twigs	*M. paniculata*	[76]
5,3′-dihydroxy-6,7,4′,5′-tetramethoxyflavone (**322**)	leaves and twigs	*M. paniculata*	[76]
5,3′-dihydroxy-7,4′,5′-trimethoxyflavone (**323**)	leaves and twigs	*M. paniculata*	[76]
5-hydroxy-7,8,3′,4′-tetramethoxyflavone (**324**)	leaves and twigs	*M. paniculata*	[76]
4′-hydroxy-5,6,7,3′,5′-pentamethoxyflavone (**325**)	leaves and twigs	*M. paniculata*	[76]
5,7,3′,4′-tetramethoxyflavanone (**326**)	leaves and twigs	*M. paniculata*	[76]
4′-hydroxy-5,7,3′-trimethoxyflavanone (**327**)	leaves and twigs	*M. paniculata*	[76]
4′-hydroxy-5, 7-dimethoxyflavanone (**328**)	leaves and twigs	*M. paniculata*	[76]
5,6,7,3′,4′,5′-hexamethoxyflavanone (**329**)	leaves and twigs	*M. paniculata*	[76]
6,7,8,3′,4′,5′-hexamethoxyflavanone (**330**)	leaves and twigs	*M. paniculata*	[76]
3-hydroxy-5,7,3′,4′-tetramethoxyflavanone (**331**)	leaves and twigs	*M. paniculata*	[76]
5,8,3′-trihydroxy-6,7,4′-trimethoxyflavone 8-*O*-β-glucopyranoside (**332**)	leaves and shoots	*M. paniculata*	[79]
5,8-dihydroxy-6,7,3′,4′-tetramethoxyflavone 8-*O*-β-glucopyranoside (**333**)	leaves and shoots	*M. paniculata*	[79]
5,7,3′,4′,5′-pentamethoxyflavanonol (**334**)	leaves and stems	*M. paniculata*	[77]
5,6,7,3′,4′,5′-hexamethoxyflavone (**335**)	leaves and stems	*M. paniculata*	[77]
5,7,8,3′,4′,5′-hexamethoxyflavone (**336**)	leaves and stems	*M. paniculata*	[77]
3,5,7,8,3′,4′-hexamethoxyflavone (**337**)	leaves and stems	*M. paniculata*	[77]
5- hydroxy-3,7,8,3′,4′-pentamethoxyflavone (**338**)	leaves and stems	*M. paniculata*	[77]
5-hydroxy-3,7,8,3′,4′,5′-hexamethoxyflavone (**339**)	leaves and stems	*M. paniculata*	[77]
8-hydroxy-3,5,7,3′,4′,5′-hexamethoxyflavone (**340**)	leaves and stems	*M. paniculata*	[77]
5,7,3′,4′,5′-pentamethoxy-flavone (**341**)	leaves	*M. paniculata*	[80]
5,7,3′,4′,5′-pentamethoxyflavanone (**342**)	leaves	*M. paniculata*	[80]
5-hydroxy-6,7,8,3′,4′,5′- hexamethoxyflavone (**343**)	leaves	*M. paniculata*	[81]
5,3′-dihydroxy-6,7,8,4′,5-pentamethoxyflavone (**344**)	leaves	*M. paniculata*	[81]
6,7,8,4′-tetramethoxy- 5,3′,5′-trihydroxyflavone (**345**)	leaves and stems	*M. paniculata*	[81]
5- hydroxy- 6,7,8,3′,4′-pentamethoxyflavone (**346**)	leaves and stems	*M. paniculata*	[81]
6,7,8,3′,4′,5′-hexamethoxyflavone (**347**)	leaves and stems	*M. paniculata*	[81]
5-hydroxy-6,7,3′,4′,5′-pentamethoxyflavone (**348**)	leaves	*M. paniculata*	[81]
5,3′- dihydroxy-6,7,4′,5-tetramethoxyflavone (**349**)	leaves and stems	*M. paniculata*	[81]
5,3′,5′-trihydroxy-6,7,4′-trimethoxyflavone (**350**)	leaves and stems	*M. paniculata*	[81]
3,5,7,3′,4′,5′-hexamethoxytlavone (**351**)	flowers	*M. paniculata*	[53]
4′-hydroxy-3,5,6,7,3′,5′-hexamethoxyflavone (**352**)	leaves	*M. paniculata*	[78]
kaempferol-3-*O*-rutinoside (**353**)	leaves and stem barks	*M. tetramera*	[82]
kaempferide-3-*O*-β-D-glucopyranoside (**354**)	leaves and stem barks	*M. tetramera*	[82]
kaempferol-3-*O*-β-D-glucopyranoside (**355**)	leaves and stem barks	*M. tetramera*	[82]
hesperitin-7-*O*-β-D-glucopyranoside (**356**)	leaves and stem barks	*M. tetramera*	[82]
neohesperidin (**357**)	leaves and stem barks	*M. tetramera*	[82]
hesperidin (**358**)	leaves and stem barks	*M. tetramera*	[82]
naringenin-7-*O*-β-D-glucopyranoside (**359**)	leaves and stem barks	*M. tetramera*	[82]
naringin (**360**)	leaves and stem barks	*M. tetramera*	[82]
rutin (**361**)	leaves and stem barks	*M. tetramera*	[82]
isoquercitrin (**362**)	leaves and stem barks	*M. tetramera*	[82]

The flavonoid glycoside types, including 5,8,3′-trihydroxy-6,7,4′-trimethoxyflavone 8-*O*-β-glucopyranoside (**332**) and 5,8-dihydroxy-6,7,3′,4′-tetramethoxyflavone 8-*O*-β-glucopyranoside (**333**), were isolated from the leaves and shoots of *M. paniculata* [79]. Furthermore, Zhou et al. [82] found the presence of ten flavonoid glycosides, encompassing kaempferol-3-*O*-rutinoside (**353**), kaempferide-3-*O*-β-D-glucopyranoside (**354**), kaempferol-3-*O*-β-D-glucopyranoside (**355**), hesperitin-7-*O*-β-D-glucopyranoside (**356**), neohesperidin (**357**), hesperidin (**358**), naringenin-7-*O*-β-D-glucopyranoside (**359**), naringin (**360**), rutin (**361**), and isoquercitrin (**362**) in the leaves and stem bark of *M. tetramera* [82] (Figure 7).

### 4.5. Terpenoids and Steroids

**Figure 8:**
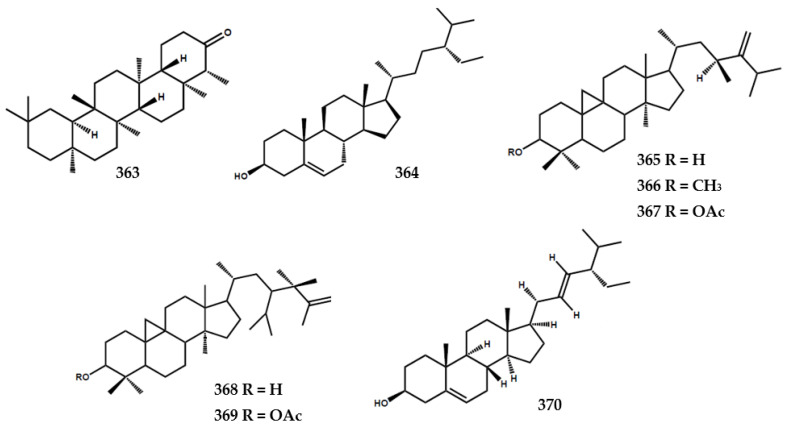
Structures of terpenoids and steroids from the *Murraya* genus **363–370**.

Terpenoids and steroids were the smallest isolated secondary metabolite group from the *Murraya* genus (Figure 8). At present, one terpenoid, namely friedelin (**363**) had been identified from the leaves of *M. euchrestifolia* [19,24]. Furthermore, steroids were rarely isolated from the *Murraya* genus, with seven compounds being identified in this review. Wu et al. [19] identified sitosterol (**364**) from *M. euchrestifolia*, and other phytosterols were isolated from the leaves of *M. exotica* [83]. These phytosterols included (23*S*)-23-ethyl-24-methyl-cycloart-24(24′)-en-3β-ol (**365**), 3β-methoxy-(23*S*)-23-ethyl-24-methyl-cycloart-24(24′)-en-3β -ol (**366**), (23*S*)-23-ethyl-24-methyl-cycloart-24(24′)-3β-yl-acetate (**367**), (23ξ)-23-isopropyl-24-methyl-cycloart-25en-3β-ol (**368**), and (23ξ)-23-isopropyl-24-methyl-cycloart-25-en-3β-yl-acetate (**369**). A previous study reported the presence of stigmasterol (**370**) in the roots of *M. gleinei* [58] and the stem bark of *M koenigii* [28] (Figure 8).

### 4.6. Other Compounds

A total of 43 compounds were identified and characterized as derivatives of alkylated and aromatic secondary metabolites (Table 4). A cyclic carotene, namely β-carotene (**371**), was isolated from the leaves of *M. euchrestifolia* along with ρ-hydroquinone (**372**) [24]. Furthermore, Barik et al. [65] discovered a new cinnamic acid derivate, namely marraxonin (**373**), from the leaves of *M. exotica*. 

A total of four new phenylpropanoid derivatives were obtained from *M. koenigii* and identified as (7′*E*,8S)-9-hydroxy-7′-propen-3′-5′-dimethoxyphenyl-3-methoxyphenyl-7,9-propane-diol-4-*O*-*β*-D-glucopyranoside (**374**), (7*R*)-2,6-dimethoxyphenyl-7,9-propane-diol-1-*O*-β-D-glucopyranoside (**375**), (2′*R*,4′*R*,7*S*)-2′,4-dihydroxy-3-methoxyphenyl-4′-hydromethyl-tetrahydro-1*H*-pyran-1-one (**376**), and (1*R*,10*S*)-1-hydroxy-7-(10-hydroxy-butyl)-2,3-dihydrobenzofuran-8(6*H*)-one (**377**) [84]. Furthermore, phenylpropanoid derivative types encompassing (1′*R*)-4-*O*-β-D-glucopyranoside-3,5-dimethoxyphenyl-1′-propanol (**378**), citrusin B (**379**), (7′*R*,8′*R*,8*E*)-4′,7′-dihydroxy-3′-methoxyphenyl-8′-hydroxymethyl-ethoxy-3,5-dimethoxyphenyl-8-propenoic acid methylester (**380**), (7*S*,8*S*)-1′-hydroxy-3′,5′-dimethoxyphenoxy-4-hydroxy-3-methoxyphenyl-7,9-propanediol (**381**), (7′*E*,7*S*,8*S*)-9′-hydroxy-7′-propen-3′-methoxy-phenyl-4-hydroxy-3-methoxyphenyl-7,9-propanediol (**382**), (1*S*,2*R*)-4,4′-hydroxy-3,3′-methoxyphenyl-1,3-propanediol (**383**), (7*S*, 8*R*)-4,4′-dihydroxy-3,3′-dimethoxyphenyl-7-ethoxy-9-propanol (**384**), (7*S*,8*R*)-4-hydroxy-3-methoxyphenyl-7,8,9-propanetriol (**385**), (7*S*,8*R*)-4-hydroxy-3,5-dimethoxy-phenyl-7,8,9-propanetriol (**386**), lariciresinol-4-O-β-D-glucopyranoside (**387**), and (7*S*,7′*S*, 8*S*,8′*R*)-4,4″,7′,9′-tetrahydroxy-3,3′,3″-trimethoxy-phenyl-7,9-propanediol (**388**) were also isolated from the species [84].

**Figure 9:**
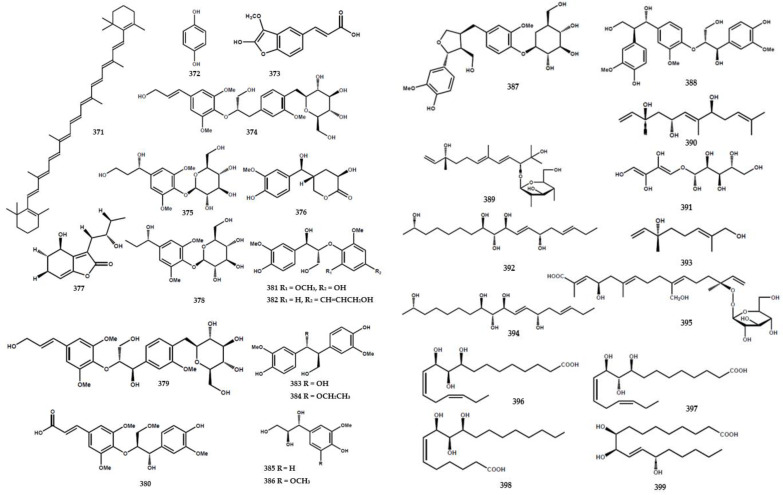

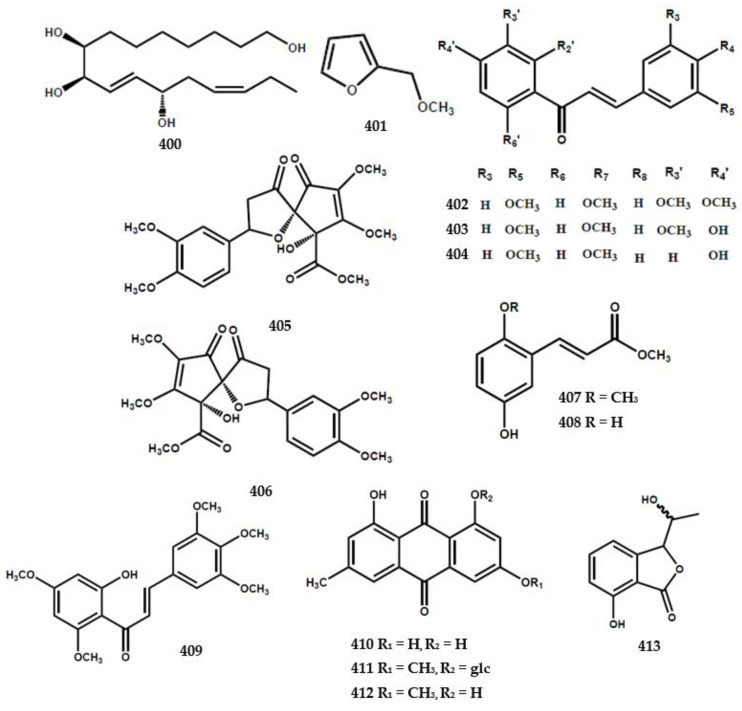
Structures of other compounds from the *Murraya* genus **371–413**.

Ma et al. [6] reported the presence of new alkene types in *M. koenigii*, including (3*S*,4*E*,6*E*,10*R*)-2,10-dihydroxy-2-hydroxy-2-methylethyl-6,10-di-methyl-4,6,11-sencolaninic-3-β-D-glucopyranoside (**389**), (3*R*,5*S*,6*E*,8*S*,10*E*)-3,7,11-trimethyl-1,6,10-dodecatriene-3,5,8-triol (**390**), (5*S*,6*R*,7*S*,8*R*)-5-amino-(2*Z*,4*Z*)-1,2,3-trihydroxybuta-2,4-dienyl-oxy-pentane-6,7,8,9-tetraol (**391**), and (3*E*,6*S*,7*E*,9*R*,10*S*,11*S*,17*R*)-octadeca-3,7-diene-6,9, 10,11,17-penta-ol (**392**) (Figure 9).

## 5. Biological Activities

*Murraya* plant parts have long been used in several regions as traditional medicines to treat dysentery, fever, and dizziness. Several studies have also shown that the extracts and compounds obtained from the genus exhibited various bioactivities, including cytotoxicity, anti-inflammatory, antidiarrheal, antihyperlipidemic, and antioxidant properties (Table 5). The active compounds have potential for medicine purposes.

### 5.1. Cytotoxicity Properties 

Ma et al. [2] reported that mahanine (**26**) showed significant cytotoxicity against four cell lines and PCK2 protein, with SPR (surface plasmon resonance) being identified as the possible mechanism. Furthermore, the potential binding sites were disclosed as Phe 525, Arg 436, Phe 530, Asn 533, and Gly 289. Changes in nuclear morphology, DNA breakage, activation-like activities, cleavage of poly(ADP-ribose) polymerase, release of cytochrome C into the cytoplasm, and stimulation of reactive oxygen species formation were observed to be signs of mahanine-induced cell death. Mahanine triggered the caspase-3, 6, 8, and 9 activities, but did not affect caspase-1-like activity [85]. Koenimbine (**58**) from *M. koenigii* showed the most potent inhibitory activity against B16 melanoma 4A5. Similar activity was also reported in Mahanimbine (**64**) and 2 other compounds (**17** and **52**) [31].

A previous study stated that three compounds from *M. Koenigii*, including pyrayafoline D, induced apoptotic cell death in HL-60 cell lines at a concentration of 30 µM. The apoptotic effect of these compounds was observed to be mediated by the loss of mitochondrial membrane potential and the subsequent activation of caspase-9/caspase-3 [32]. Furthermore, CHCl_3_ extract and koenoline (**80**) from *M. koenigii* exhibited cytotoxic activity with an ED_50_ range of 4.0 µg/mL to 26 µg/mL [38]. 

The primary screening results showed that compounds derived from *M. siamensis* had inhibitory activity. All test coumarins obtained through isolation showed a potent dose-dependent inhibitory effect on EBV-EA induction via TPA. Murrayacoumarin A (**304**) bearing an oxygenated geranyloxy side chain exhibited the most potent activity [74].

Ma et al. [27] reported several compounds from *M. koenigii*, including mahanine (**26**), mahanimbine (**16**), and 8,8′-biskoenigine (**71**), that showed significant PTP1B inhibitory activity with IC_50_ values of 1.773, 1.875, and 2.286 µM, respectively.

**Table 5:** Biological activities from the *Murraya genus*.

Biological Activities	Cell Target/Process	Compounds or Extract [IC_50_/CD_50_]	Plant Species	References
Cytotoxic	Du145, HepG2, HeLa, and HCT-116 cell lines	murrayamine A (**24**) [0.3 ± 0.4 µM; 3.4 ± 0.3 µM; 0.4 ± 1.7 µM; 0.2 ± 0.4 µM]; mahanine (**26**) [2.2 ± 0.1 µM; 3.5 ± 0.9 µM; 0.02 ± 0.01 µM; 0.03 ± 0.08 µM];	*M. microphylla*	[2]
	HepG2, Du145, HeLa, and HCT116 cell	murrayamine A (**24**) [21.4 ± 3.1 µM; 19.7 ± 1.1 µM; 25.9 ± 3.7 µM; 20.0 ± 2.3 µM]; mahanine (**26**) [48.3 ± 3.4 µM; 46.9 ± 2.5 µM; 46.5 ± 0.2 µM; 44.8 ± 3.2 µM];	*M. microphylla*	[29]
	inhibited melanogenesis B16 melanoma 4A5	murrayamine-E (**17**) [2.9 µM]; mahanimbicine (**52**) [2.2 µM]; koenimbine (**58**) [1.2 µM]; mahanimbine (**64**) [1.4 µM];	*M. koenigii*	[31]
	induced apoptosis in HL-60 cells through activation of the caspase-9/caspase-3 pathway	pyrayafoline D (**54**); murrafoline I (**90**),	*M. koenigii*	[32]
	HepG2 cells	(−)-bispyrayafoline C (**114**); (+)-bispyrayafoline C (**115**); kwangsine D (**122**); kwangsine E (**123**); kwangsine G (**125**); kwangsine H (**126**); kwangsine J (**128**); kwangsine K (**129**); kwangsine L (**130**); kwangsine M (**131**); euchrestine C (**135**); 1-hydroxy-3-methyl-9H-carbazole (**137**); murrayafoline A (**146**) [Range 9.9–44.3 µM]	*M. kwangsiensis*	[3]
	HL-60 and HeLa	murrastinine-C (**87**) [17 µg/mL and 1 µg/mL]; murrayatanine-A (**88**) [12 µg/mL and 5 µg/mL];	*M. koenigii*	[39]
	KB cell culture	CHCl_3_ extract; koenoline (**80**); murrayanine (**98**)	*M. koenigii*	[38]
	bearing an oxygenated geranyloxy side-chain exhibited the most potent activity	murrayacoumarin A (**304**)	*M. siamensis*	[74]
	PTB1B inhibitory	mahanine (**26**), mahanimbine (**16**), and 8,8′-biskoenigine (**71**)	*M. koenigii*	[27]
Anti-inflammatory	inhibitory activities against NO production	3-formylcarbazole (**35**) [78.2 ± 2.6 µM]; *O*-demethylmurrayanine (**83**) [79.2 ± 2.1 µM]; murrayanine (**98**) [12.2 ± 0.2 µM]; 1-methoxy-3-(methoxymethyl)-carbazole (**140**) [65.1 ± 1.7 µM];	*M. kwangsiensis*	[3]
	potent inhibition against LPS-induced NO production in BV-2 microglial cells	panitin D (**283**) [19.6 ± 0.3 µM];; exotimarin I (**287**) [26.9 ± 0.8 µM]; trans-dehydroosthol (**290**) [12.4 ± 0.9 µM];	*M. paniculata*	[57]
	inhibitory effects on LPS-induced NO production in BV-2 microglial cells	2′-*O*-ethylmurrangatin (**204**) [53.2 ± 8.9 µM]; panitin C (**231**) [57.7 ± 5.8 µM]; exotimarin H (**232**) [53.2 ± 4.4 µM];	*M. paniculata*	[59]
	inhibition of NO production	murratin D (**219**) [39.0 ± 4.3 µM]; muratin E (**220**) [36.8 ± 3.4 µM]; muralatin C (**229**) [32.7 ± 3.0 µM]; 2-(7-methoxy-2-ocochromen-8-yl)-3-methylbut-2-enyl)-3-methylbut-2-enoate (**230**) [38.1 ± 3.0 µM]; exotimarin H (**232**) [28.6 ± 0.9 µM];	*M. exotica*	[56]
	inhibitions against LPS-induced NO production in RAW264.7 macrophages	(2′*R*,4′*R*,7*S*)-2′,4-dihydroxy-3-methoxyphenyl-4′-hydroxymethyl-tetrahydro-1*H*-pyran-1-one (**376**) [32.7 µM]; (1*R*,10*S*)-1-hydroxy-7-(10-hydroxybutyl)-2,3-dihydrobenzofuran-8(6*H*)-one (**377**) [7.9 µM]; (7′*E*,7*S*,8*S*)-9′-hydroxy-7′-propen-3′-methoxyphenyl-4-hydroxy-3-methoxy-phenyl-7,9-propanediol (**382**) [42.1 µM]; (7*S*,8*R*)-4,4′-dihydroxy-3,3′dimethoxy-phenyl-7-ethoxy-9-propanol (**384**) [58.9 µM]; lariciresinol-4-O-*β*-D-glucopyranoside (**387**) [62.4 µM];	*M. koenigii*	[84]
Hepatoprotective and Antihyperlipidemic	against D-galactosamine induced HL-7702 cells damage (hepatoprotective) the activations of PPARα and PPRγ (Antihyper-lidemic)	(1′*R*,3′*R*,4′R,6′S)-endocycliomurrayamine A (**31**); claulansiums A (**42**); 1′-*O*-methyl-claulamine B (**44**); dunnine E (**45**) 3-formyle-7-hydroxy-9*H*-carbazole-1-O-β -D-glucopyranoside (**32**); 4′-hydroxy-phenyl-6ethyl-1*H*-pyrrole-2-carbax-aldehyde (**33**); pyrolezanthine (**37**); portulacatone (**41**)	*M. koenigii*	[4]
	inhibited nitric oxide production in BV-2 microglial cells stimulated with lipopolysaccharide	murradiate (**192**) and murradiol (**193**)	*M. tetramera*	[54]
Antidiarrheal	inhibitory activity against castor-oil-induced diarrhea and PGE2-induced enteropooling in rats	kurryam (**57**); koenimbine (**58**)	*M. koenigii*	[5]
Antioxidant	antioxidative activities DPPH method	(3*S*,4*E*,6*E*,10*R*)-2,10-dihydroxy-2-hydroxy-2-methylethyl-6,10-di-methyl-4,6,11-sencolaninic-3-β-D-gluco-pyranoside (**389**) [38.4 µM]; (3*R*,5*S*,6*E*,8*S*,10*E*)-3,7,11-trimethyl-1,6,10-dodecatriene-3,5,8-triol (**390**) [23.5 µM]; (3*E*,6*S*,7*E*,9*R*,10*S*,11*S*,17*R*)-octadeca-3,7-diene-6,9,10,11,17-pentaol (**392**) [25.4 µM]; capsianoside V (**395**) [40.2 µM];	*M. koenigii*	[6]

### 5.2. Anti-Inflammatory Properties

Studies on the biological activities of plants from the *Murraya* genus identified the presence of anti-inflammatory activity. Furthermore, Murrayanine (**98**) from *M. kwangsiensis* showed significant inhibition of NO production in lipopolysaccharide-stimulated BV-2 microglial cells compared to a positive control [3]. Another study reported that three compounds from *M. paniculata*, including Panitin D (**283**), exotimarin I (**287**), and trans-dehydroosthol (**290**), showed moderate inhibitory effects on LPS-induced NO production in BV-2 microglial cells [57].

### 5.3. Hepatoprotective and Antihyperlipidemic Properties

Hepatoprotective properties refer to the ability of a substance to prevent damage to the liver. Previous studies reported that CHCL_3_ extract from *M.* koenigii exhibited potential hepatoprotective properties. Furthermore, four compounds, encompassing (1′*R*,3′*R*,4′*R*,6′*S*)-endocycliomurrayamine A (**31**), claulansiums A (**42**), 1′-*O*-methylclaulamine B (**44**), and dunnine E (**45**), showed moderate activity against D-galactosamine-induced toxicity in HL 7720. Antihyperlipidemic agents were substances known to promote the reduction of lipid and cholesterol levels. Several studies showed that compounds from *Murraya* exhibited moderate activity [4].

### 5.4. Antidiarrheal Properties

Mandal et al. [5] reported that kurryam (**57**) and koenimbine (**58**) exhibited significant inhibitory activity against castor-oil-induced diarrhea and PGE_2_-induced enteropooling in rats. Furthermore, a dose of 30 mg/kg had an equivalent effect to 5 mg/kg of the standard drug.

### 5.5. Antioxidant Properties 

Previous studies on the bioactivity of the *Murraya* genus showed potent antioxidant activity. According to a previous report, (3*S*,4*E*,6*E*,10*R*)-2,10-dihydroxy-2-hydroxy-2-methylethyl-6,10-di-methyl-4,6,11-sencolaninic-3-β-D-glucopyranoside (**389**), (3*R*,5*S*,6*E*, 8*S*,10*E*)-3,7,11-tri-methyl-1,6,10-dodeca-triene-3,5,8-triol (**390**), (3*E*,6*S*,7*E*,9*R*,10*S*,11*S*,17*R*)-octadeca-3,7-diene-6,9,10,11,17-pentaol (**392**), and capsianoside V (**395**) from *M. koenigii* showed the potent inhibition of DPPH with an IC_50_ range of 21.4–49.5 µM [6].

## 6. Conclusions

In conclusion, *Murraya* species have been extensively studied, thereby contributing to the understanding of secondary metabolites and their biological activities in nature. Furthermore, alkaloids were observed to be the dominant compounds from *Murraya,* followed by coumarins and flavonoids. The literature reports showed that the genus exhibited various biological activities, such as cytotoxic and anti-inflammatory effects.

## Figures and Tables

**Table 4:** Other compounds from the *Murraya genus*.

Compounds	Part of Plant	Source	References
β-carotene (**371**)	leaves	*M. euchrestifolia*	[24]
ρ-hydroquinone (**372**)	leaves	*M. euchrestifolia*	[24]
marraxonin (**373**)	roots	*M. exotica L.*	[65]
(7′*E*,8*S*)-9′-hydroxy-7′-propen-3′,5′-dimethoxyphenyl-3-methoxy-phenyl-7,9-propanediol-4-O-β-D-glucopyranoside (**374**)	whole plant	*M. koenigii*	[84]
(7*R*)-2,6-dimethoxyphenyl-7,9-propanediol-1-O-β-D-glucopyranoside (**375**)	whole plant	*M. koenigii*	[84]
(2′*R*,4′*R*,7*S*)-2′,4-dihydroxy-3-methoxyphenyl-4′-hydroxymethyl-tetrahydro-1H-pyran-1-one (**376**)	whole plant	*M. koenigii*	[84]
(1*R*,10*S*)-1-hydroxy-7-(10-hydroxybutyl)-2,3-dihydrobenzofuran-8(6H)-one (**377**)	whole plant	*M. koenigii*	[84]
(1′*R*)-4-O-β-D-glucopyranoside-3,5-dimethoxyphenyl-1′-propanol (**378**)	whole plant	*M. koenigii*	[84]
citrusin B (**379**)	whole plant	*M. koenigii*	[84]
(7′*R*,8′*R*,8*E*)-4′,7′-dihydroxy-3′-methoxyphenyl-8′-hydroxymethyl-ethoxy-3,5-dimethoxyphenyl-8-propenoic acid methylester (**380**)	whole plant	*M. koenigii*	[84]
(7*S*,8*S*)-1′-hydroxy-3′,5′-dimethoxyphenoxy-4-hydroxy-3-methoxyphenyl-7,9-propanediol (**381**)	whole plant	*M. koenigii*	[84]
(7′*E*,7*S*,8*S*)-9′-hydroxy-7′-propen-3′-methoxyphenyl-4-hydroxy-3-methoxyphenyl-7,9-propanediol (**382**)	whole plant	*M. koenigii*	[84]
(1*S*,2*R*)-4,4′-hydroxy-3,3′-methoxyphenyl-1,3-propanediol (**383**)	whole plant	*M. koenigii*	[84]
(7*S*,8*R*)-4,4′-dihydroxy-3,3′-dimethoxyphenyl-7-ethoxy-9-propanol (**384**)	whole plant	*M. koenigii*	[84]
(7*S*,8*R*)-4-hydroxy-3-methoxyphenyl-7,8,9-propanetriol (**385**)	whole plant	*M. koenigii*	[84]
(7*S*,8*R*)-4-hydroxy-3,5-dimethoxyphenyl-7,8,9-propanetriol (**386**)	whole plant	*M. koenigii*	[84]
lariciresinol-4-O-β-D-glucopyranoside (**387**)	whole plant	*M. koenigii*	[84]
(7*S*,7′*S*,8*S*,8′*R*)-4,4″,7′,9′-Tetrahydroxy-3,3′,3″-trimethoxyphenyl-7,9-propanediol (**388**)	whole plant	*M. koenigii*	[84]
(3*S*,4*E*,6*E*,10*R*)-2,10-dihydroxy-2-hydroxy-2-methylethyl-6,10-di-methyl-4,6,11-sencolaninic-3-b-D-glucopyranoside (**389**)	whole plant	*M. koenigii*	[6]
(3*R*,5*S*,6*E*,8*S*,10*E*)-3,7,11-trimethyl-1,6,10-dodecatriene-3,5,8-triol (**390**)	whole plant	*M. koenigii*	[6]
(5*S*,6*R*,7*S*,8*R*)-5-amino-(2Z,4Z)-1,2,3-trihydroxybuta-2,4-dienyloxy-pentane-6,7,8,9-tetraol (**391**)	whole plant	*M. koenigii*	[6]
(3*E*,6*S*,7*E*,9*R*,10*S*,11*S*,17*R*)-octadeca-3,7-diene-6,9,10,11,17-pentaol (**392**)	whole plant	*M. koenigii*	[6]
(2*E*,6*R*)-2,6-dimethyl-2,7-octadiene-1,6-diol (**393**)	whole plant	*M. koenigii*	[6]
(6*R*,7*E*,9*S*,10*R*)-6,9,10-trihydroxy-7-octadecenoic acid (**394**)	whole plant	*M. koenigii*	[6]
capsianoside V (**395**)	whole plant	*M. koenigii*	[6]
(9*S*,10*R*,11*R*,12*Z*,15*Z*)-9,10,11-trihydroxy-octadeca-12,15-dienoic acid (**396**)	whole plant	*M. koenigii*	[6]
oxylipin (**397**)	whole plant	*M. koenigii*	[6]
(8*R*,9*R*,10*S*,6*Z*)-trihydroxyoctadec-6-enoic acid (**398**)	whole plant	*M. koenigii*	[6]
(9*S*,10*R*,11*E*,13*S*)-9,10,13-trihydroxyoctadec-11-enoic acid (**399**)	whole plant	*M. koenigii*	[6]
(8*S*,9*R*,10*E*,12*S*,14*Z*)-heptadeca-10,14-diene-1,8,9,12-tetraol (**400**)	whole plant	*M. koenigii*	[6]
ferulyl esters (**401**)	whole plant	*M. omphalocarpa*	[48]
2′-hydroxy-3,4,4′,6′-tetramethoxychalcone (**402**)	leaves and twigs	*M. paniculata*	[76]
2′-hydroxy-3,4,3′,4′,6′-pentamethoxychalcone (**403**)	leaves and twigs	*M. paniculata*	[76]
2′,4-dihydroxy-3,5,4′,6′-tetramethoxychalcone (**404**)	leaves and twigs	*M. paniculata*	[76]
(+)-murrayaspiroketone (**405**)	leaves and stems	*M. paniculata*	[59]
(−)-murrayaspiroketone (**406**)	leaves and stems	*M. paniculata*	[59]
methyl 2-methoxy-5-hydroxycinnamate (**407**)	leaves	*M. paniculata*	[67]
methyl 2,5-dihydroxycinnamate (**408**)	leaves	*M. paniculata*	[67]
2′-hydroxy-3,4,5,4′,6′-pentamethoxychalcone (**409**)	leaves	*M. paniculata*	[80]
emodin (**410**)	leaves and stem barks	*M. tetramera*	[82]
emodin-8-*O*-β-D-glucopyranoside (**411**)	leaves and stem barks	*M. tetramera*	[82]
Physcion (**412**)	leaves and stem barks	*M. tetramera*	[82]
3ξ-(1ξ-hydroxyethyl)-7-hydroxy-1-isobenzofuranone (**413**)	stem bark	*M. koenigii*	[28]

## Data Availability

The study did not report any data.
